# Improving Gram Stain Sensitivity in the Diagnosis of Septic Arthritis

**DOI:** 10.7759/cureus.97552

**Published:** 2025-11-23

**Authors:** Daniel Jones, Grace White

**Affiliations:** 1 Trauma and Orthopaedics, Bradford Royal Infirmary, Bradford, GBR

**Keywords:** culture, gram-stain, sensitivity, septic arthritis, specificity

## Abstract

Background and hypothesis

Gram staining is a fundamental part of the early diagnosis of septic arthritis and helps direct initial antimicrobial therapy. Though research shows uniform high specificity, its sensitivity is low with a comparatively wide spread of values. We hypothesise that a significant component of the variation in Gram stain sensitivity may be attributable to suboptimal laboratory technique and limited experience.

Method

This is a retrospective observational study. Data from a total of 269 native and prosthetic joint aspirates were collected over two 12-month periods: October 2017 to October 2018 (cohort 1) and January 2023 to January 2024 (cohort 2). Between these periods, the Gram staining technique remained the same; however, the laboratory achieved accreditation from the United Kingdom Accreditation Service (UKAS). This accreditation was achieved through improved staff training in Gram staining technique, and a policy of second opinions for challenging samples was introduced. Data were analysed using MS Excel (Microsoft Corporation, Redmond, Washington, United States) and StatsKingdom software.

Results

Following UKAS accreditation, Gram stain sensitivity improved from 25.6% to 44.7%. A one-tailed proportions test demonstrated this to be statistically significant at p = 0.035. Regarding secondary outcomes, *S. aureus* was the most prevalent organism. It was cultured in isolation or as a polymicrobial finding in 51% of aspirates in cohort 1 and in 34% of samples in cohort 2. Crystal arthropathy and septic arthritis were not mutually exclusive. In cohort 1, 12% crystal arthropathy cases also grew microorganisms. In cohort 2, 15% of crystal arthropathies also had a positive culture.

Conclusion

In keeping with previous work, this study also shows Gram staining has relatively low sensitivity in the diagnosis of septic arthritis. However, retrospective analysis shows that significant improvements can be made through training and supportive laboratory practices and may go some way to explain the variation in Gram stain sensitivity in the wider literature.

## Introduction

This is a retrospective observational study that explores whether laboratory technician skill and training explain some of the variability in Gram stain sensitivity in the diagnosis of septic arthritis and provides a rationale for future prospective research. Septic arthritis is the inflammation of a joint due to an infectious aetiology. Locally, it may cause rapid and irreversible destruction of a joint and systemically, septic shock. With a mortality of 11.5% and morbidity of 31.6%, prompt and accurate diagnosis is of great importance, though this remains a significant challenge [1]. Gram stain specificity for septic arthritis has repeatedly been shown to be high, between 97% and 100% [2-6]. In contrast, sensitivity is relatively low, and the wider literature demonstrates a greater spread of values, 17%-45% [2-6]. The reasons for this spread in values are not well-explored.

Gram staining was first developed in 1884 and still remains a key technique in the diagnosis of a wide range of bacterial infections [7]. The technique first involves smearing a sample of bacteria on a slide, drying, and fixing the smear with heat, which is followed by the application of crystal violet dye, which is taken up by the bacterial cell wall and fixed with iodine. Second, a solvent such as ethanol is applied, which removes the stain from the lipid-rich cell walls of Gram-negative bacteria, while Gram-positive organisms retain the violet colour. A final fuchsin stain is applied to better identify the decolourised Gram-negative organisms [8]. It can be performed quickly and helps determine the presence, absence, and basic characteristics of microorganisms.

When investigating how the sensitivity of Gram staining may be improved, one may consider the arthrocentesis (joint aspiration) or the laboratory assessment. Good arthrocentesis practice involves thorough skin decontamination, a sterile field, and personal protective equipment. Arthrocentesis plausibly has a substantial role to play in Gram stain specificity and false negatives [9]. Though once a sample is obtained, the arthrocentesis technique likely has minimal impact on sensitivity. Gram stain technique and interpretation require skill and experience to reveal microorganisms; where this is lacking, false negatives and low sensitivity may occur [8]. 

## Materials and methods

Study design

This is a retrospective observational analysis of the outcomes of synovial fluid Gram staining in the investigation of septic arthritis. Data from a total of 269 native and prosthetic joint aspirates were collected over two 12-month periods: October 2017 to October 2018 (cohort 1) and January 2023 to January 2024 (cohort 2). Between these periods, Gram stain methodology fundamentally remained unchanged; however, the laboratory achieved accreditation from the United Kingdom Accreditation Service (UKAS). UKAS accreditation requires external reviews of the technical competence of staff, quality assurance of lab processes, such as sample handling, and validity of testing methods [10]. The key changes that achieved this accreditation were as follows: increased utilisation of second opinions. This was introduced in two forms, firstly, an encouragement to seek advice in hours for challenging samples. Secondly, digital images of slides were saved to allow for second opinions where staffing did not permit immediate review. Only those samples where there was doubt regarding the result underwent a second opinion. Data for the exact numbers of Gram stain identified by second opinion was not available. Urgent joint fluid samples were also prioritised to minimise delay in analysis. Extensive training and re-training were also introduced, and competence following this was assessed as part of performance appraisals. 

Inclusion and exclusion criteria

Between October 2017 and October 2018 (cohort 1), 121 patients underwent synovial fluid analysis for suspected septic arthritis of both native and prosthetic joints. Two patients in cohort 1 were excluded due to sample contamination. Contamination was defined as the identification of a microorganism on culture or polymerase chain reaction (PCR) that was not treated as septic arthritis with antibiotics and surgical washout. Between January 2023 and January 2024 (cohort 2), 148 patients underwent joint aspiration for suspected septic arthritis; four patients were excluded due to sample contamination. 

Data collection

All patients referred for septic arthritis at a UK district general hospital were logged on an electronic referrals form, and patient data from these forms were cross-referenced with Gram stain and microbial culture results on the Integrated Clinical Environment (ICE) system.

Data analysis

A one-tailed proportions test was used to assess if there was a significant difference in Gram stain sensitivity between cohort 1 and cohort 2. Statskingdom.com software was used for the calculation. A diagnosis of septic arthritis was defined as the presence of microorganisms on culture or PCR in conjunction with a documented clinical diagnosis and treatment. 

## Results

Of the 119 patients in cohort 1 included in the final analysis, 43 had positive cultures; of these, 11 had a preceding positive Gram stain. Gram stain sensitivity for this period was 25.6% and specificity was 100% (Table 1). In cohort 2, 144 patients were included in the final data set; 38 had positive cultures, and of these, 17 had a preceding positive Gram stain. Sensitivity of Gram staining increased to 44.7% and specificity remained at 100% (Table 2). A one-tailed proportions test demonstrated a significant improvement in Gram stain sensitivity (p = 0.035) following implementation of lab reforms. 

**Table 1:** Gram stain analysis of synovial fluid from October 2017 to October 2018 (cohort 1)

Field	Value
Total of patients included	119
Total of positive Gram stains	11
Total of positive cultures	43
Gram stain sensitivity	25.6%
Gram stain specificity	100%

**Table 2:** Gram stain analysis of synovial fluid from January 2023 to January 2024 (cohort 2)

Field	Value
Total of patients included	144
Total of positive Gram stain	17
Total of positive cultures	38
Gram stain sensitivity	44.7%
Gram stain specificity	100%

**Figure 1:**
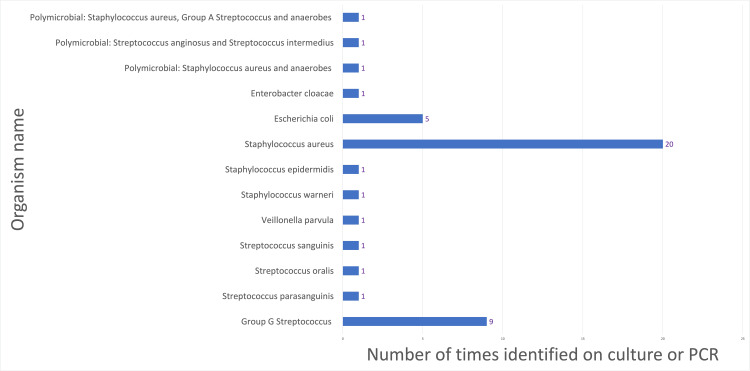
Organisms identified from synovial fluid from October 2017 to October 2018 (cohort 1) Bar chart illustrating the count of different organisms identified following synovial fluid sampling from a total of 119 patients

**Figure 2:**
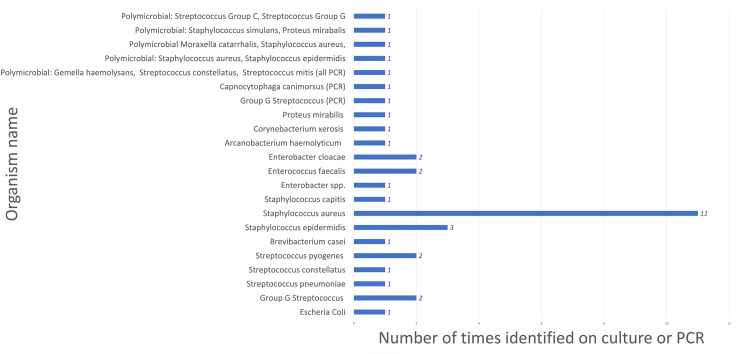
Organisms identified from synovial fluid analysis from January 2023 to January 2024 (cohort 2) Bar chart illustrating the count of different organisms identified following synovial fluid sampling from a total of 144 patients

*Staphylococcus aureus *(*S. aureus*) was the most common organism identified in both cohort 1 and cohort 2 (Figures 1, 2). Representing an isolated or polymicrobial finding in 51% of aspirates in cohort 1 and in 34% of samples in cohort 2. 

## Discussion

Following UKAS accreditation, brought about by the introduction of reforms to Gram stain training and easy access to second opinions, there was a significant improvement in Gram stain sensitivity from 25.6% to 44.7% (p = 0.035). Therefore, skill and training in the nuances of Gram staining may explain some of the variation in its sensitivity in this hospital and possibly in the wider literature. 

Considering lab factors that may influence false negatives, the following may all prevent the identification of an organism before the stain is applied. An initial smear was made too thick, with excessive heat during fixing and failure to adequately dry the initial smear before stain application. During the application of stains and counterstains, there are further stumbling blocks that may prevent microorganism identification. These are the following: low concentrations of the initial crystal violet, insufficient iodine to fix the crystal violet, and prolonged use of the decolourising solvent and excessive counterstaining [8]. In addition, the lab in this study also noted that samples can simply fail to stick to slides, and cellular debris may be mistaken for microorganisms to a less-trained eye. One can therefore appreciate the value of training that focuses on these challenges and the utility of second opinions.

False negatives and low sensitivity could also be driven by factors outside of the lab, such as concurrent antibiotics. Antibiotics are routinely withheld until aspirates are taken unless a patient is septic. In this study, an accurate assessment of whether antibiotics were withheld until completion of synovial fluid sampling was not possible. Antibiotic administration times are precisely logged on an electronic medication administration chart. In contrast, documentation of arthrocentesis is done in retrospect, and the specific time of the procedure is rarely written. Perhaps paradoxically, concurrent antibiotic therapy may not have a bearing on Gram stain results. A 500-patient cohort study in Switzerland included 105 patients under concurrent antibiotic therapy and found no significant correlation with Gram stain outcome [11]. Similarly, one might postulate that concurrent antibiotic therapy would likely affect both Gram stain and culture in equal measure and so have little effect on the correlation of Gram stain and culture [5]. 

The age of a culture is another modifiable factor that can affect Gram stain results. Older samples are prone to degradation of the peptidoglycan walls that hold the initial violet stain and so may appear as Gram-negative [8]. Though in this study, all Gram stains correlated with their subsequent culture. 

Other changes in laboratory practice might also improve Gram stain outcomes. In this study, the Gram staining technique remained fundamentally the same; however, modified techniques show promise in improving the distinction between Gram-positive and Gram-negative organisms [12,13]. This, in turn, might help direct early antibiotic therapy and improve morbidity and mortality. Early differentiation between the two groups is key. Empirical treatment in the UK is typically flucloxacillin, which lacks Gram-negative cover, yet up to 20% cases may be driven by Gram-negative organisms [14-16]. In cohort 1, 16% of cultures identified Gram-negative species in isolation or as a polymicrobial finding and 21% in cohort 2.

Further advancements in synovial fluid analysis include PCRs. PCR has greater sensitivity than traditional culture and may also rapidly identify resistance markers. Nevertheless, PCR is recommended as an adjunct to Gram stain and culture and not a replacement [17]. In cohort 2, PCR identified organisms in three samples that were missed by Gram stain and culture. PCR was not used for the 2017-2018 data set. 

Additional secondary findings are also broadly in keeping with the wider literature. A 2025 UK multicentre analysis of septic arthritis aspirates found that *S. aureus* was the most common organism, accounting for 37% [2]. Similarly, this study found that *S. aureus* was most prevalent. It was cultured in isolation or as a polymicrobial finding in 51% of aspirates in cohort 1 and in 34% of samples in cohort 2. These data also support the notion that crystal arthropathy and infection are not mutually exclusive. In cohort 1, 12% crystal arthropathy cases also grew microorganisms. In cohort 2, 15% of crystal arthropathies also had a positive culture. 

Aside from synovial fluid analysis, a range of other tools may be used to aid in septic arthritis diagnosis, though each has its drawbacks. Kocher’s criteria are a clinical prediction tool with up to 99.6% sensitivity that is well-validated in children, though not in adults [18,19]. It makes use of non-weight-bearing status, pyrexia, raised white cell count, and erythrocyte sedimentation rate [18,19]. Caird et al. developed this further with the addition of CRP > 20 mg/L as a key predictor [20]. X-ray and ultrasound may demonstrate the presence of an effusion, but cannot determine if it is sterile or not. Increased bone oedema on MRI appears to be more common in septic arthritis, compared with mimics such as gout and transient synovitis; however, overall, MRI has poor specificity [21,22]. 

Limitations and recommendations

As a small single-centre study, the external validity of this data is not certain. However, in centres where Gram stain sensitivity is low, reforms focused on training, re-training, and easy access to second opinions in the laboratory are likely beneficial. The data set available for this study lacks in-depth data in two key areas: the number of septic arthritis cases identified by second opinion, and there is an absence of a reproducible assessment of Gram stain competence. Nonetheless, these data provide a strong rationale for a larger prospective study, and future analysis should address these two issues. A structured assessment might include analysis of a lab technician's Gram stain technique, their ability to critique another's technique, and an exam featuring images of challenging Gram stain slides. Challenging slides should feature common pitfalls that might affect interpretation, such as cellular debris, samples taken following antibiotic administration, and older samples. 

## Conclusions

In keeping with previous work, this study shows that Gram staining has relatively low sensitivity for the diagnosis of septic arthritis. Gram staining is a nuanced and skilled technique; improved training and access to second opinions significantly improve its sensitivity and may go some way to explain the wide variation in Gram stain sensitivity for septic arthritis diagnosis in the wider literature. A prospective study would provide more robust evidence and give a more detailed framework of how similar results might be achieved in other laboratories.
